# Characteristics and Genetic Diversity of Local Populations of Giant Spiny Frog (*Quasipaa spinose*)

**DOI:** 10.3390/genes17040411

**Published:** 2026-03-31

**Authors:** Zhi-Hui Zhu, Miao-An Shu

**Affiliations:** Key Laboratory of Freshwater Aquaculture and Breeding, College of Animal Sciences, Zhejiang University, Hangzhou 310058, China

**Keywords:** *Quasipaa spinose*, genetic structural variation, SNP genotyping, pan-genome analysis, immunity

## Abstract

Background/Objectives: To establish a foundation for conserving and utilizing local frog germplasm resources in Zhejiang Province, for *Quasipaa spinose,* which has high commercial and nutritional value, a pan-genome analysis was performed. Methods: Herein, we characterized 405,263 SNPs for the giant spiny frog, *Q. spinose*, using the Illumina NovaSeq platform. Results: These loci were highly polymorphic in 59 individuals sampled from three different subpopulations, with 0.05 to 0.30 minor alleles per locus. The observed and expected heterozygosities were 0.2379 and 0.2683 (IBD), respectively. These polymorphic loci would be useful for assessing genetic diversity, population structure, gene flow, population assignment, and paternity in giant spiny frogs. Conclusions: Our investigation demonstrated that there are distinct genetic and evolutionary histories between Zhejiang and Jiangxi frogs. Phylogenetic inference effectively differentiated these three subpopulations based on their geographical origins, and the phylogenetic inference level of domesticated Zhejiang frogs was comparatively higher than that of the Jiangxi-derived population. Furthermore, by utilizing three selective signature methods, namely, Obs/Exp het, nucleotide diversity (Pi), and identical by state (IBS), across subpopulations, we concluded that these three breeds were from an identical population, and no genetic bottleneck occurred among these three lineages, in accordance with LD decay analysis. Finally, 2700 potential candidate genes were identified, including MAPK, calcium signaling pathway, Ras signaling pathway and regulation of actin cytoskeleton; we noted that the key genes associated with dilated cardiomyopathy or arrhythmogenic right ventricular cardiomyopathy in humans beings and GnRH signaling pathway-related genes (i.e., CD80, IFNA, and KCNK1) were highly enriched, which could impact cardiac function through immune-associated genes.

## 1. Introduction

The giant spiny frog, *Quasipaa spinose*, is a threatened species with high commercial and nutrition value [1,2]. With the expansion of artificial breeding, the phenomenon of homozygosity has become severe, leading to declining immunity, malformation, deceleration of individual growth, and so on [3]. Therefore, the study of the genetic diversity of local populations and the genetic structure of the giant spiny frog is indispensable [4,5,6,7] facilitating the development of breeding and culture [8]. Additionally, amphibians have been identified as a good model for studying the factors that shape patterns of genetic variation and differentiation [9,10,11], which is attributable to their limited dispersal ability and high levels of population genetic structuring compared to other animal classes [12,13,14]. The Chinese spiny frog, an amphibian native to southern China and northern Vietnam, has been threatened by recent infectious disease outbreaks caused by bacterial, viral, and parasitic infections, which severely impact the sustainable development of the *spinose* farming industry [15,16,17]. Therefore, the selective breeding of *Q. spinose* is required, especially given the relationship of its nutritional value with its genetic characteristics.

Wei et al. [18] examined the microsatellite diversity in *Q. spinose*, where the phylogeographic studies were initially based on mitochondrial DNA/mtDNA [18]. Taking advantage of the mitochondrial 12S rRNA gene, Wu Rui-qiong identified two major populations of *Q. spinose* in Fujian Province; additionally, SNP loci in the 12S rRNA gene in the two major populations were screened for rapid specific SNP genotyping [19,20]. Given the well-known evolutionary inferences based on mtDNA, next-generation sequencing (NGS) methods have largely replaced mtDNA and microsatellite markers in phylogeography and population genetic investigations of non-model organisms [21]. However, there has been a noticeable lack of genome research specifically focusing on the *spinose* breeds, particularly the development and selection processes of giant spiny frogs. Additionally, the landscape of structural variation (SV) in breeds has not yet been thoroughly explored or documented, let alone its influence on complex straits. Thus, our study will provide a closer control population, allowing more accurate inference of genetic characteristics of *spinose*.

In this study, we applied the Illumina NovaSeq platform to conduct genotyping and genetic evolution analysis on three *Q. spinose* breeds. Using the P-distance matrix, we created a phylogenetic tree. Finally, three breeds of *Q. spinose*, consisting of two domesticated and one wild population, were separated into two groups, and the selective sweeps inferred were used to screen for candidate genes connected with immunity, i.e., CD88, CD80, IFNA and eutD, all of which are involved in cardiomyopathy-associated signaling pathways in human beings. Our results exhibited strong selection signals in both SNPs and SVs. Overall, our study provides a genetic resource database for selective breeding and highlights the importance of structural variations in studying genome structure and complex traits among breeds.

## 2. Materials and Methods

### 2.1. Ethics Statement

This study was accomplished in strict compliance with the Guidelines and Policies (GBT42011-2022; GBT39760-2021; GBT35892-2018; GBT39852-2008) of the Biological Studies Animal Care and Use Committee, Laboratory Animal Center, Zhejiang University, People’s Republic of China.

### 2.2. Animal Sample Collection and Morphologic Calculation

The samples of *Q. spinose* were collected from Jiande, Hangzhou; Ningdu, Jiangxi; and Longquan, Lishui. In total, we obtained 59 samples from three local cities, and ~20 adult specimens per site were collected. Muscle tissue was collected from the posterior limb and preserved in a −80 °C superfreezer. SampleInfo was annotated as HZ_1~20, JX_1~20, LS_1~19. As for the value of the body mass index (BMI) calculation, we refer to the formulate of BMI = body mass (g)/ height^2^ (cm^2^), with three decimal points.

### 2.3. Genotyping and Quality Control (QC)

Muscle genomic DNA was extracted using the standard phenol–chloroform protocol. The extracted DNA samples were then amplified over the whole genome. VAHTSTM DNA Clean Beads (no manufacturer required) were used to purify the PCR product; 400 bp insert fragments were selected for paired-end sequencing, utilizing the paired-end (PE) 2 × 150 bp mode of Illumina NovaSeq. The program bwa (0.7.12-r1039) [22] mem was applied with default parameters; high-quality reads were then mapped onto the reference genomic database. The software samtools (1.16.1) [23] was used to rearrange and transverse sam into bam files. Finally, all of the acquired SNP data were checked using gatk (version: 3.7-0-gcfedb67) HaplotypeCaller and VariantFiltration [22,23,24]. The parameters were as follows: --filterExpression “QD < 2.0 || FS > 60.0 || MQ < 40.0 || SOR > 6.0 || QUAL < 30.0 || MQRankSum < −12.5 || ReadPosRankSum < −8.0”. The quality control was as follows: (1) sample detection rate > 0.95, (2) SNP detection rate > 0.95, (3) minor allele frequency (MAF) > 0.05, and (4) Hardy–Weinberg equilibrium (HWE) *p*-value > 1 × 10^−6^. The results were visualized using snpEff (version: snpEff_v4_5covid19) and vcftools (version: 0.1.16) [6]. SNP loci with 20% missing were utilized in LD decay [12,14], PCA [25], phylogenetic tree construction [26], analysis of genetic structure [27], Gmatrix [28], and identical by state (IBS) analysis. As a representative case, we simplified the procedure of a typical genome-wide association study, and the data including visualized schematic graphs were all provided by PersonalBio Tech, Shanghai, China (Project Number: PN20251109027) [29,30].

### 2.4. Analysis of Population Genetic Characteristics

Following quality control and DNA sequence alignment by bwa and Picard, we conducted a further analysis of population genetic variation to clarify the molecular mechanism underlying the selective evolution. (1) Principal component analysis (PCA) was completed with plink v1.9 program.VCF2D v0.1.17 [12] was utilized to compute the P-distance matrix, which serves as the foundation for ATGC: FastTree [31] to construct a Neighbor Joining Tree (NJ tree). Genetic population structure was analyzed by Admixture software 1.3.1 with the K values ranging from 2 to 10. In this study, the optimal K value was chosen based on the lowest Cross-Validation (CV) value. (2) LD decay analysis: the LD coefficient (*r*^2^) was calculated for each pair of SNPs, with a maximum distance of 5000 kb between them. PopLDdecay v3.42 [32] was used to generate LD decay graphs according to the distance between SNPs [30]. (3) Selection sweep methods: the fixation index (F_st_), cross-population extended haplotype homozygosity (XP-EHH), and nucleotide diversity (PI) analysis were combined, to detect selective signatures between and within populations. We annotated the loci or windows within the 1% and 5% thresholds with reference to the *Q. spinose* genome (Taxonomy ID: 109965). Ultimately, a common set was obtained by intersecting the annotated genes [32].

### 2.5. Enrichment Analysis of Candidate Genes

Gene Ontology (GO) was conducted by topGO [33], followed by numbering the listed genes for the annotated terms; the *p* value was calculated by a 4D dimensional distribution program, uncovering highly enriched GO terms; and the principal biological functions were determined in sequence. The top GO enrichment was cataloged into molecular function (MF), biological process (BF) and cellular component (CC); the top 10 GO terms are exhibited in the text. Meanwhile, Kyoto Encyclopedia of Genes and Genomes (KEGG) analysis was performed by the standard procedure, with genes within a 5% threshold via the online tool DAVID. Terms and pathways with a *p*-value < 0.05 were considered significantly enriched. The enrichment results were visualized using ggplot2 in R (R version 2.15.1 (2012-06-22), x86_64-unknown-linux-gnu).

## 3. Results

### 3.1. Geographic Range

**Figure 1:**
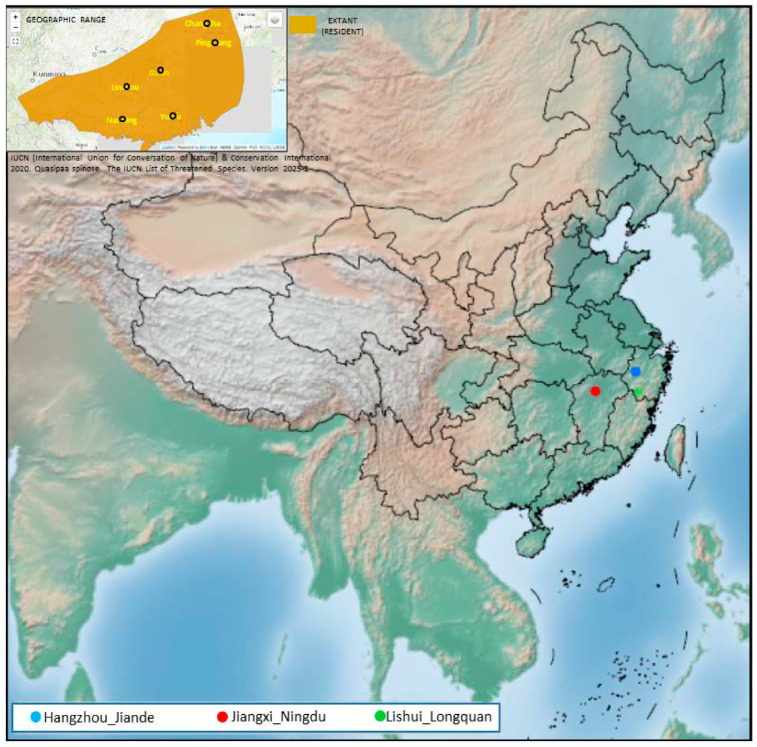
Three population sites of 59 individuals of *Q. spinose* from central and east China (black symbols). The gray shade in the left corner insert indicates the entire species distribution range, downloaded from the IUCN website. The phylogenetic tree in the right corner insert is a phylogenic tree constructed in MEGA X; branch labels represent bootstrap support value. The three genetic lineages are indicated with different colors, which are also used in all other figures. The samples were named according to their geographic sites, as follows: HZ_1~20 (Hangzhou_Jiande), JX_1~20 (Jiangxi_Ningdu), and LS_1~19 (Lishui_Longquan).

The Chinese spiny frog has most recently been listed in the IUCN Red List as vulnerable under criteria A4cd in 2019 because of a suspected population decline of 30% over a three-generation period between 2009 and 2024, which is believed to be caused from over-harvesting and ongoing habitat destruction and loss [8]. This species was previously thought to range from China to Vietnam. However, it is now thought to be Chinese-endemic [20]. It is found in central, southwestern and southern China, including Hong Kong. The distribution of this species was previously thought to be more widely distributed in Yunnan, Guizhou and Zhejiang Provinces; however, some of these records were previous misidentifications, and it is not thought to occur as widely in these areas (Z. Yuan pers. comm. June 2019). Following morphological analysis, a subpopulation from Che-ki in Anhui Province, China, has now been assigned to *Quasipaa courtoisi*, which has been resurrected from synonymy in this species [4]. It is not expected to occur more widely in China, as it is replaced by its congeners outside of its distribution (Z. Yuan pers. comm. June 2019) (www.iucnredlist.org. (Accessed: 7 December 2023)). It was previously reported from Vietnam; however, there has been longstanding confusion, which was noted in the 2004 assessment, over these records. Many older records of this species refer to other taxa in the genera, including *Paa verrucospina* and *P. yunnanensis*. Bourret (1942: 291) stated that he encountered this species only in Mao Son and Bac-Kan, and there have been more recent reports from Vietnam including Ha Giang Province [7]. However, all records of this species from northeastern Vietnam are now recognized as *Q. acanthophora* (A. Ohler pers. comm. March 2021) (Figure 1).

### 3.2. Selective Gross Morphologic Variation in Q. spinose

**Figure 2:**
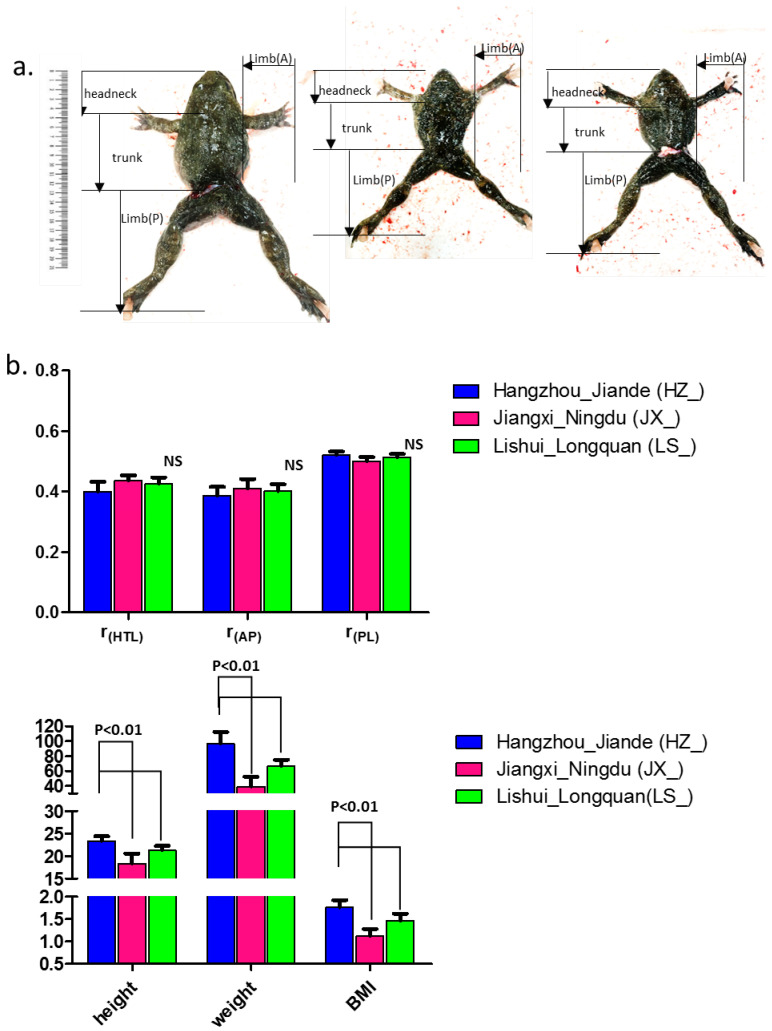
The phenotypic variation among the three *Q. spinose* breeds. (**a**) Measurement of the mean weight and height of *Q. spinose*; compared BMI (body mass index); the color of dorsal skin visualization of the three genetic lineages; (**b**) One-way ANOVA (*p* < 0.05) was used for statistical analysis. NS: no significance; r_(HTL)_: ratio of head to trunk length; r_(AP)_: ratio of anterior to posterior limb length; r_(PL)_: ratio of posterior limb length to height.

We grouped 59 individuals according to their genetic clusters (Figure 2a). For each collected sample, we compared the body mass index (BMI) to the nearest value of 1.755, and measured the height and weight of the individual to the nearest quantity of 23.382 cm and 96.53 g, respectively (Figure 2b). We also compared the mean limb length of individuals using a parametric ANOVA, as these traits were normally distributed (Figure 2b). The statistics analysis showed that the BMI of Jiangxi_Ningdu’s frogs was dramatically declined, from 1.755 to 1.116, with significance of *p* < 0.01; however, the ratio of posterior limb to body length was biased to the ratio of 0.501 with no significance, indicating that the population from Jiangxi_Ningdu seemed much slimmer. According to the economic efficiency, the quantity and flavor of limb muscle from *Q. spinose* from Hangzhou_Jiande is more satisfying. Thus, this is a taxonomically diagnostic characteristic used to demarcate different subspecies. And we reached the conclusion that *Q. spinose* from Hangzhou_Jiande, representative of Zhejiang Province, comprehensively satisfied the trait requirements in our study for our selective breeding goal (Figure 2).

### 3.3. Sequencing and SNP Calling

DNA samples from 59 individuals of *Q. spinose* were sequenced using an Illumina Novaseq, generating a total of 1.34 billion paired-end reads. There were 18.26 billion bases with a quality score of at least 30 (Q30) after mapping to the reference genome (Taxonomy ID: 109965), and the guanine–cytosine content was 44.69%. The mapping rate reached 99% among three geographically derived populations of *Q. spinose*. We obtained approximately 12.41 million tags in total, and their average sequencing depth was less than 1. A total of 1.13 million biallelic SNPs were annotated by the snfEff software [21,24] (Supplementary Table S1).

**Figure 3:**
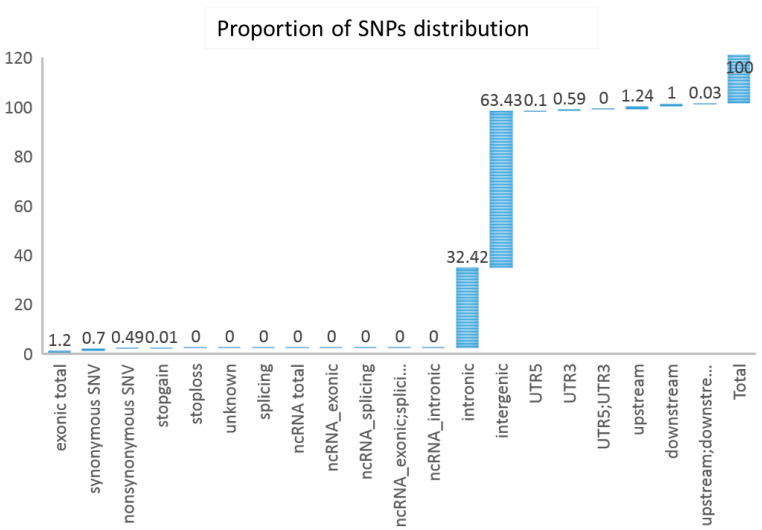
The annotation of SNPs in *Q. spinose*. The schematic represents the statistics analysis of the number of each type of mutation.

After filtering, the dataset contained 405,263 SNPs for LD decay analysis, phylogeny and structure analysis, Gmatrix, and IBS analysis [34]. The distribution of LD decay showed that the three subpopulations had high genetic diversity, good representativeness of species, and historical demography, which were called for in our genetic structural analysis (Figure 3, Supplementary Figures S1 and S2).

### 3.4. Genetic Structure Analysis

**Figure 4:**
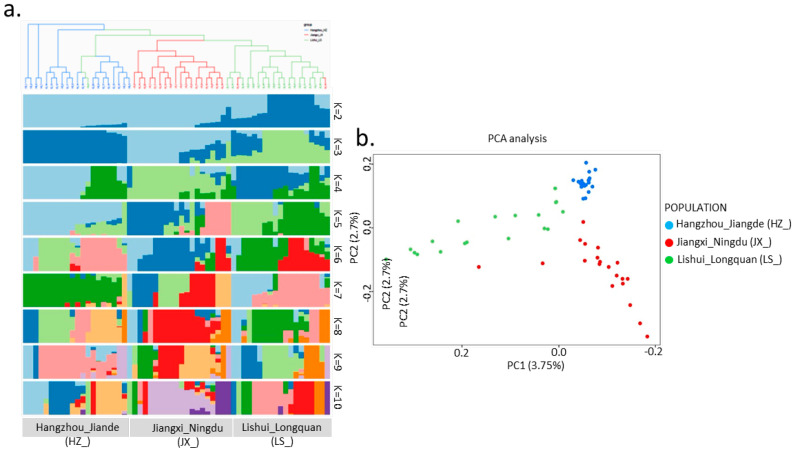
Inferred genetic structure of *Q. spinose* population according to LD decay determining the performance of genetic structure analysis, with its advantage of high genetic diversity, good sample collection and identical genetic background, (**a**) Bayesian cluster analysis using fastSTRUCTURE from K = 2 to K = 10 based on the 405,263 SNP dataset, and (**b**) PCA based on a 405,263 SNP dataset. In (**a**), codes above and below the plot refer to population and cluster identifiers, respectively. Different clusters are indicated with different colors In (**a**), the different color of individual data points is coded according to their cluster identities.

Principal component analysis (PCA) based on 405,263 SNPs showed that three geographic subpopulations formed three independent clusters, determining that genetic differentiation occurred among the three subpopulations, and geographic isolation had resulted in structural diversity in genomic respects. In detail, we found that Hangzhou_Jiande frogs had a relatively low level of genome differentiation, featured in convergent plots in the PCA diagram, compared to the other two groups; the frogs from Lishui_Longquan had much higher heterogeneity according to their extent in axis PC1, from the left-most to the central area, while the genetic diversity of Jiangxi_Ningdu breeds occurred in the middle of the other two, characterized with red dots showing less tightness and much more extension in axis PC2 (Figure 4 and Supplementary Figure S2).

### 3.5. Phylogenetic Analysis

**Figure 5:**
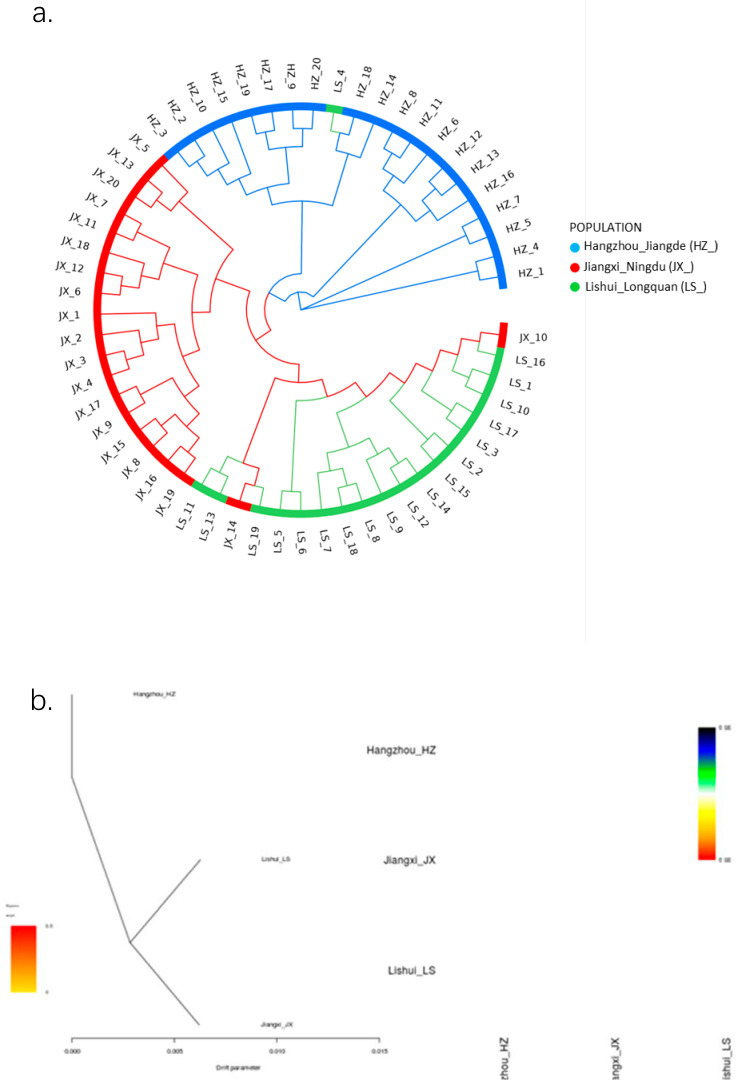
(**a**) Phylogenic tree of three populations of giant spiny frog (*Q. spinose*) breeds, with blue representing Hangzhou_Jiande (HZ_), red representing Jiangxi_Ningdu (JX_), and green representing Lishui_Longquan (LS_). The phylogenic tree suggests that the root of the tree is differentiated into two parts: one (upper right) represents the population of Hangzhou (blue symbol), and the other major clade (lower left) represents the population of Jiangxi and Lishui (red and green symbols); (**b**) the further extension of interior minor clades differentiates the red subpopulation (JX_) from the green one (LS_).

Phylogenetic analysis based on 405,263 SNPs revealed three major clusters in the phylogenetic tree (Figure 5), concordant with geography and the PCA results. Compared to Hangzhou_Jiande frogs, Jiangxi_Ningdu frogs exhibited a closer genetic distance with Lishui_Longquan’s frogs. From the admixture analysis depicted in Figure 4, we ascertained that the experimental CV value reached its minimum when K = 3 (optimized value is suitable for biological expectation and sample collection), indicating a distinct differentiation among these three subpopulations. When K ranged from 2, 4 to 10, Jiangxi_Ningdu frogs exhibited nearly identical ancestral components, standing apart from Lishui_Longquan frogs. Integrally, it represents three geographic subpopulations of *Q. spinose* and the cluster from Hangzhou_Jiande (HZ_) well, representing populations located in Zhejiang Province when K = 3, while the cluster from Lishui_Longquan (LS_) is much closer to the central cluster from Jiangxi_Ningdu (JX_). However, the time axis for evolution in *Q. spinose* is not yet documented in the text (Figure 5).

### 3.6. Molecular Diversity and Selective Sweep

To screen candidate genes associated with the diversity of traits among the population lineages of *Q. spinose*, two groups were categorized: Group1 encompassed two from Lishui_Longquan and Hangzhou_Jiande, individually; Group2 encompassed one from Jiangxi_Ningdu. We then made use of three methods, fixation index (Fis), cross-population extended haplotype homozygosity (XP-EHH), and nucleotide diversity (PI/π), to assess genetic diversity between and within subpopulations. We found that the values of three parameters from those subpopulations were quite close, and the variation in observed and expected heterozygosity among clusters was considerable, ranging from 0.228 to 0.246 for observed heterozygosity and from 0.266 to 0.277 for expected heterozygosity; however, the Obs Het values were all lower than Exp Het, while the value of Fis fell into the range of 0.12~0.15, suggesting that the genetic diversity had declined, which might be attributed to intra-breeding, selective propagation, and/or disequilibrium population structure. The level of Pi was a bit high (approximately 0.28) in three lineages of *Q. spinose*, suggesting that variation in these loci was not rare. Conclusively, there was no occurrence of extremely hard bottleneck reactions among the subpopulations, which led to a severe decline in genetic diversity; this determinant was in accordance with the characteristics of previous LD decay analysis.

**Figure 6:**
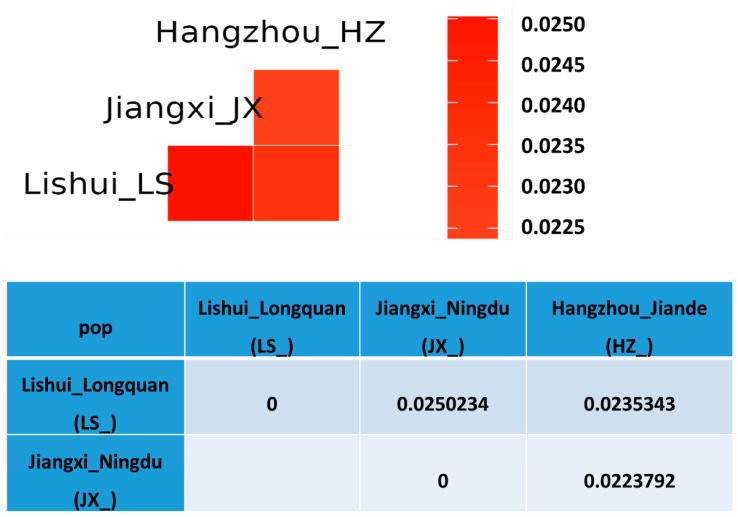
Molecular diversity and selective sweep of *Q. spinose*. The heatmap illustrates the genetic distance among 3 genetic lineages, including Lishui_Longquan (LS_), Jiangxi_Ningdu (JX_) and Hangzhou_Jiande (HZ_). The maximum value of F_st_ was detected between JX_ and LS_. The θπ plotted diagram serves as another index.

Pairwise F_st_ values were all statistically significant, with an average pairwise F_st_ = 0.0236456, ranging from 0.0223792 to 0.0250234, demonstrating that Jiangxi_JX and Lishui_LS have the largest genetic differentiation, followed by Lishui_LS and Hangzhou_HZ group, while Jiangxi_JX was the closest to Hangzhou_HZ with the smallest F_st_ value (0.0223792). The relatively large F_st_ for Jiangxi_JX vs. Lishui_LS suggested a severe geographic isolation and/or a large geographic distance, leading to poor gene flow between these two subpopulations. In summary, *Q. spinose* populations are geographically highly structured, and the results from the fastSTRUCTURE, PCA, and phylogenetic analysis suggest that the three *Q. spinose* subpopulations sampled from southeast and east China are three separate geographically and genetically distinct lineages (Figure 6, Supplementary Figures S4 and S5).

### 3.7. GO and KEGG Enrichment

**Figure 7:**
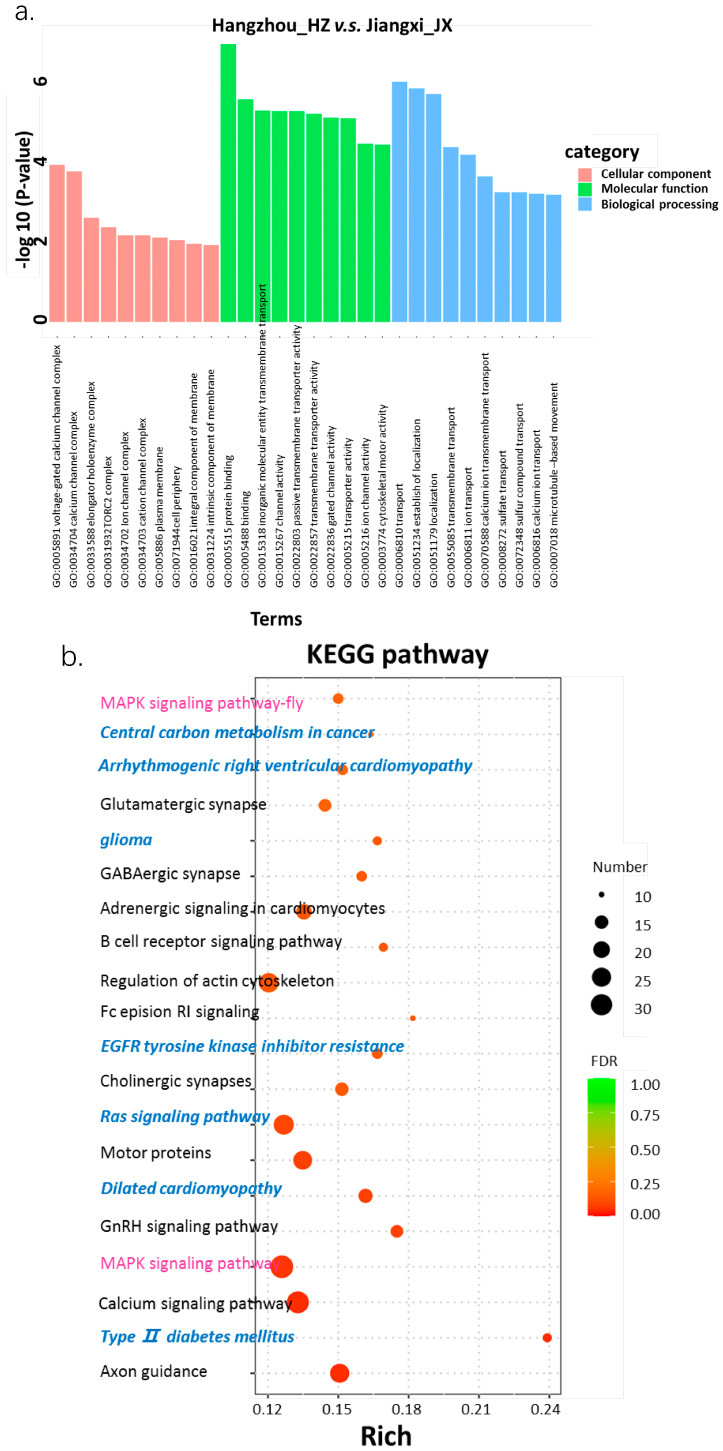
(**a**) GO and (**b**) KEGG analysis for enrichment of candidate genes. The significant KEGG pathway enrichment of the candidate genes under selection in *Q. spinose*. Hangzhou_Jiande (HZ_) vs. Jiangxi_Ningdu (JX_) KEGG pathways were analyzed via KEGG database (https://www.kegg.jp/kegg/kegg1.html) (accessed on 6 February 2026).

Lastly, enrichment analyses of candidate genes using Gene Ontology (GO) and Kyoto Encyclopedia of Genes and Genomes (KEGG) analysis were performed within a 5% threshold via the online tool DAVID; 2700 genes were identified by GO and KEGG enrichment. In the GO enrichment analysis, a total 30 significantly enriched terms (*p* < 0.05) were uncovered, distributed among cellular components, molecular function, and biological processing. An intriguing phenomenon was that a countable number of these terms were linked to channel complex and activity and cellular compound transportation, encompassing genes such as TBXT, PDGFD, and EGFR [7,35,36,37]. Those genes were always associated with adipose biosynthesis in populations of *Q. spinose*, as well as in sheep breeding [7]. The KEGG analysis revealed 20 significantly enriched pathways, including innate immunity, embryonic development, disease-associated pathology, such as the B cell receptor signaling pathway, MAPK, calcium signaling, cardiomyopathy and Type II diabetes mellitus disease in human beings, encompassing immune-associated genes CD88, CD80, IFNA and eutD. Conclusively, we deduced that the JX_ population was always suffering from environmental stressors, not facilitating their habitation, but in the state of high sensitivity to stressors, which was attributed to their ‘slimness’. In comparison, the climate [31,38] and geography in Zhejiang are much warmer, allowing farmers of giant spiny frogs to enjoy more economically advantageous breeding conditions and economic rewards (Figure 7).

## 4. Discussion

We investigated the genetic diversity of the giant spiny frog (*Q. spinose*), a representative of the amphibians, noted for its ectothermic nature, highly permeable skin [39], and complex life cycle. Its survival, growth, activity and development are strongly dependent on specific temperature ranges, which are fundamentally affected by climate change. We hypothesized that different geographic populations might have distinctive phenotypes, such as the frog’s body mass index (Figure 2) and the nutritional value and flavor of its muscle (i.e., posterior limb muscle) [1,2].

Leveraging pan-genome SNP molecular markers, this study assessed the population genetic structure of three different *Q. spinose* populations. The three breeds were divided into two subgroups based on the PCA results. That is because Jiangxi_Ningdu (JX_) is closer to wild species (no data shown), while Lishui_Longquan (LS_) and Hangzhou_Jiande (HZ_) are closer to bred ones. Notable differences in their genomes suggest that the agricultural climate of Jiangxi has fostered distinct frog breeds that are distinguishable from those in Lishui and Hangzhou in Zhejiang Province, demonstrating that the geographic climate is readily impacting the development of *Q. spinose* during its life cycle.

We established an NJ tree for the three breeds based on the P-distance matrix.The clustering of *Q. spinose* breeds at a common node suggests genetic proximity among them. However, compared to samples from Hangzhou_Jiande, the NJ tree reveals a closer distance between Lishui_Longquan and Jiangxi_Ningdu. Both LS_ and JX_, originating from HZ_, exhibit a closer kinship with the HZ_breed, which indicates that the difference ecosystems between the western and southwestern regions of Zhejiang have led to differences among the *Q. spinose* populations, implying that the geographic isolation is stronger, prohibiting them from propagation. Theoretically, intercrossing between JX_ and LS_, accelerating the heterogeneity rate, will represent an optimized proposal for giant spiny frog breeding, while F_st_ is smallest with the value of 0.022, suggesting the impacts of inbreeding.

The admixture analysis demonstrated that Zhejiang *Q. spinose* frogs possess fundamentally distinct ancestral components from their foreign counterparts. The Lishui_Longquan and Hangzhou_Jiande breeds manifest a more diverse ancestry when compared to Jiangxi_Ningdu’s *Q. spinose*, suggesting that the unique geographical and climatic conditions in Jiangxi may have imposed certain limitations on frog migration and admixture, thereby causing a relatively homogenous genetic background for the local frog population. Consequently, there is a need for proactive efforts in introducing foreign genetics resources and fostering increased genetic exchange among different frog populations. The discovery that LS_ and JX_lineages share similar ancestral components and can be distinguished from the HZ_lineage when K = 3 may stem from historical hybridization events. JX_ is a breed developed through careful breeding over years from the crossbreeding of LS_ with local frogs in Zhejiang, which may have contributed to its divergence from other frog breeds of *Q. spinose* (Figure 5).

Population genetic analysis was able to distinguish physiological differences and geographical origins. In this study, HZ_ and LS_ breeds came from Zhejiang Province; however, their genetic difference was in the minimum of the range of differentiation (pairwise F_st_ = 0.023), and similar results were seen from PCA and fastSTRUCTURE (Figure 4b and Figure 6). One reason is that Hangzhou is located in the north and Lishui is located in the southwest of Zhejiang Province; however, the climate in Jiande and Longquan belongs to the typical subtropical season type. The terrain of Zhejiang Province slopes step-like from the southwest to the northeast, with mountainous and hilly areas in the southwest (Longquan, Lishui). A second explanation is attributed to the fact that the HZ_ breed was hybridized with native frog populations. Interestingly, we found four clustered in a branch of the lineage (Figure 5). However, Lishui belongs to the Zhejiang group; according to the PCA figures, it is closer to the JX_ breed than the HZ. In the fastSTRUCTURE result when K = 3, the breed appears to have some introgression of JX_ breed (Figure 4a), which needs to be confirmed by more evidence. Overall, these data provide strong evidence of the genetic structure of these three *Q. spinose* breeds. Overall, we could suppose that heterozygotic inter-breeding was the premier proposal, accompanied by heightened heterozygosity between subpopulations; meanwhile, the impact of inbreeding was emphasized, especially in the pairwise Jiangxi and Hangzhou group.

Apart from the deep genetic divergences revealed by both phylogenetic analysis and low pairwise F_st_ values, the three lineages exhibited clear differentiation in phenotypic traits. Although differentiation in the mean values of partially studied traits, i.e., height, weight, and pigment, was significant, morphological variation suggested that divergence in body mass index (height, weight) was probably driven by selection. Therefore, it was noteworthy that the magnitude of phenotypic differentiation (P_st_) exceeded, on average, that of neural genetic differentiation (F_st_), suggesting that the possibility of environmentally induced difference cannot be excluded as an alternative explanation for divergence in the value of BMI. Since the neutral expectation is that P_st_ should be equivalent to F_st,_ any deviation from this calls for an explanation. Similarly, the fact that phenotypic differentiation was a negative function of neutral differentiation is noteworthy. The mechanistic explanation for this negative correlation is that some of the most genetically divergent populations were phenotypically the least diverged (e.g., lineages HZ_ and JX_ in Figure 2).

Without common garden data, we cannot conclusively establish an ultimate explanation for the observed patterns. Nevertheless, we suspected that the divergence in BMI may be related to different sexual selection regimes in different lineages, as observed in other systems [34,35,40,41,42]. Hence, an interesting avenue for future studies of reverse genetics would be to investigate whether the phenotypic differentiation in body mass index might act as prezygotic isolation mechanism among the three genetically divergent lineages.

After conducting the enrichment analysis, 30 terms and 20 pathways were selected. We assessed whether the voltage-gated calcium channel complex or calcium channel complex, as a representative, was significantly distinguished in CC (cellular component) terms, which is in accordance with the differentiated molecular function terms, such as transporter activity and channel activity. As for biological process terms, we still paid attention to transport terms, such as GO:0022857 transmembrane transport, ion transport and calcium ion transport. Meanwhile, major pathways, through the KEGG database, were associated with innate immunity, responding to the stressors from the internal and external environment. Although the lineage of giant spiny frogs from Jiande, Hangzhou city, might undergo adaptive immunity and (or) have chronic pathological status, such as Type II diabetes mellitus, which always confers an overwhelming anabolism, the economic worth attributed to agricultural breeding is still promising.

In conclusion, our current work determined that the breed from Hangzhou city, Zhejiang Province, could be a germplasm resource for selection during selective breeding of *Q. spinose*, excluding its muscular quantity and quality, but our limited knowledge of its molecular mechanisms still calls for further investigation.

## 5. Conclusions

In this investigation, we found that there are genetic structural variations among three lineages in *Q. spinose*; a foundation was established for conserving and utilizing local frog germplasm resources in Zhejiang Province; dramatic significant selective signatures were collected through molecule diversity analysis; and sweeping and enrichment amplified selective areas for evolution, such as immunity, which opened up access for colleagues to explore genetic diversity through SNP sequencing in *Q. spinose* and can provide local farmers with more guidance in breeding.

## Data Availability

The study data are available on request from the corresponding author.

## Supplementary Materials

The following supporting information can be downloaded at: https://www.mdpi.com/article/10.3390/genes17040411/s1. Figure S1. High quality of sequencing data. Figure S2. Admixture analysis. Figure S3. The distribution diagram of LD decay. Figure S4. The genetic evolution of three subpopulations. Figure S5. The plotted diagram of selective sweeping. Table S1. SNP calling and mapping.
