# Supplementary material for: Characteristics and Genetic Diversity of Local Populations of Giant Spiny Frog (Quasipaa spinose)
Source: Genes (Basel). 2026 Mar 31;17(4):411. doi: 10.3390/genes17040411 (PMC13116599; doi:10.3390/genes17040411)
Supplement: Supplementary file 1 [file genes-17-00411-s001.zip › genes-4169579-supplementary.pdf]

Supplementary Materials

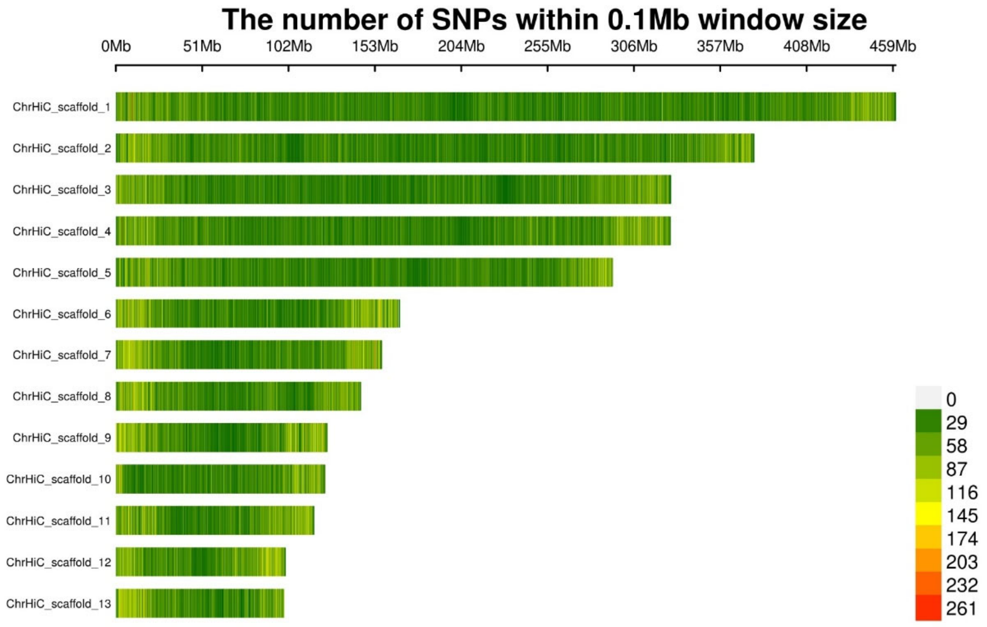

The number of SNPs within 0.1Mb window size
0Mb
51Mb
102Mb
153Mb
204Mb
255Mb
306Mb
357Mb
408Mb
459Mb
ChrHiC_scaffold_1
ChrHiC_scaffold_2
ChrHiC_scaffold_3
ChrHiC_scaffold_4
ChrHiC_scaffold_5
ChrHiC_scaffold_6
ChrHiC_scaffold_7
ChrHiC_scaffold_8
ChrHiC_scaffold_9
ChrHiC_scaffold_10
ChrHiC_scaffold_11
ChrHiC_scaffold_12
ChrHiC_scaffold_13
0
29
58
87
116
145
174
203
232
261

(a)

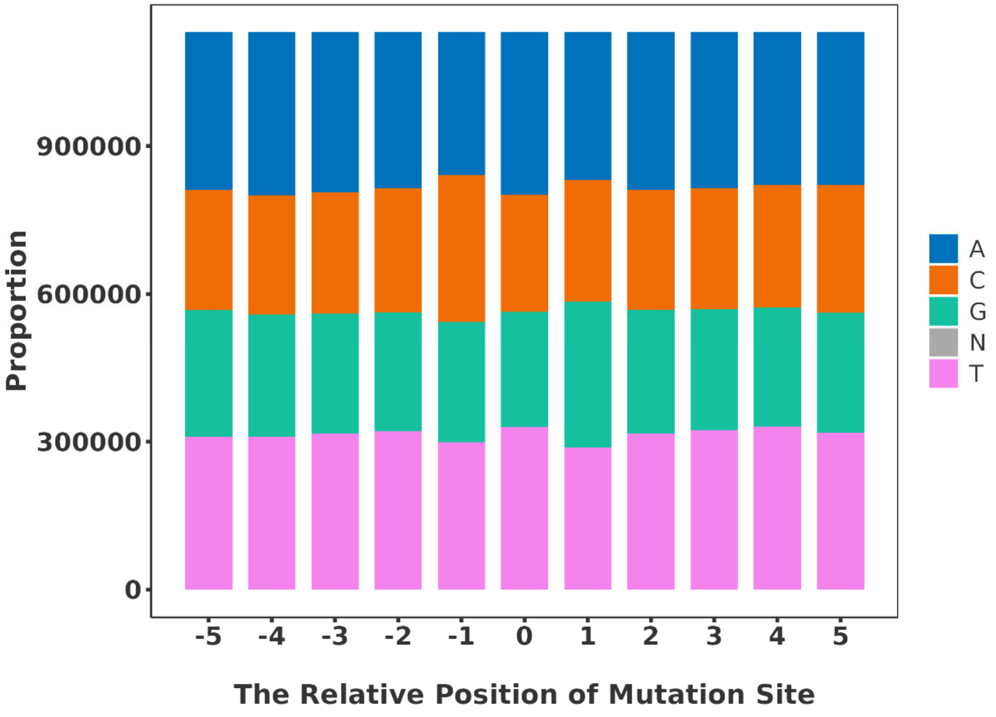

Proportion
900000
600000
300000
0
-5
-4
-3
-2
-1
0
1
2
3
4
5
The Relative Position of Mutation Site
A
C
G
N
T

(b)

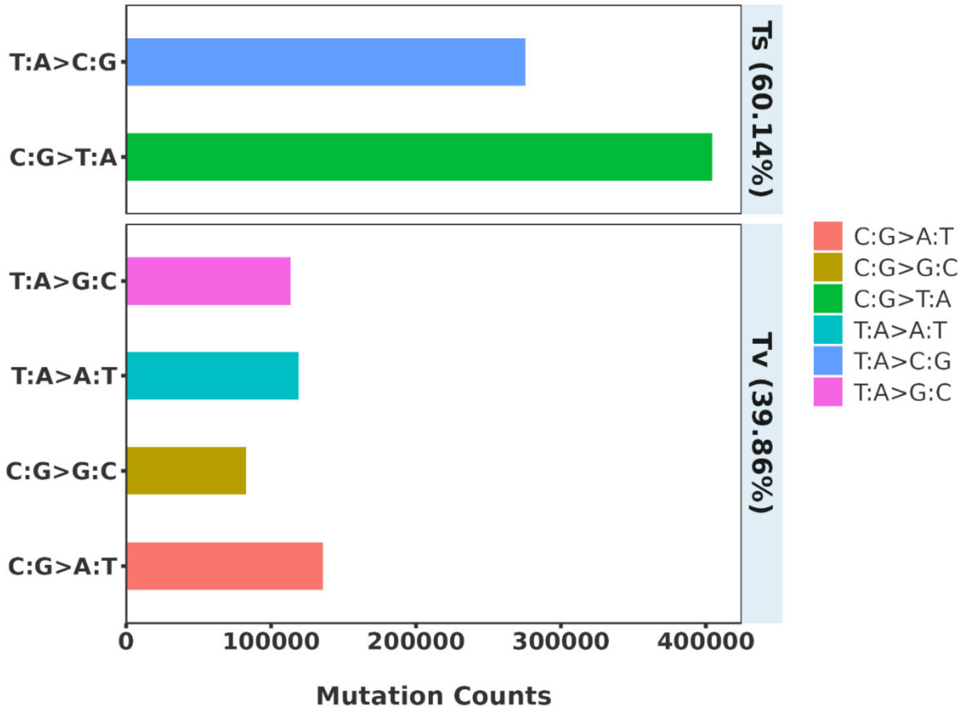

(c)

Figure S1. High quality of sequencing data. (**a**) The distribution of SNPs in each chromosome in *Quasipaa spinose*, which is called for the next population structure analysis; (**b**) the statistics analysis of bias of mutation loci; the horizontal axis indicates the position of mutation site, and the vertical axis indicates the number of mutations of each base. Zero represents the mutation loci; the negative value is the base in front of the loci; the position value is the base behind of the loci (**c**) the frequency of mutation in SNP detection.

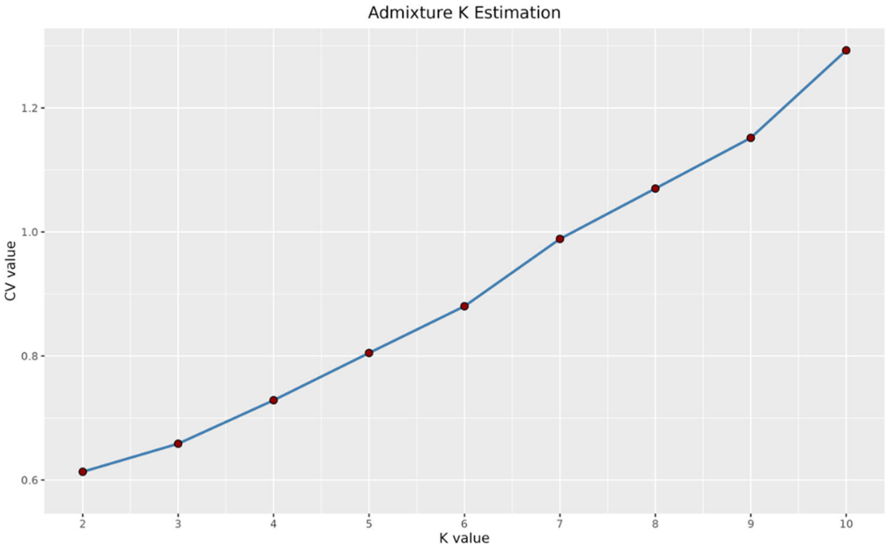

Figure S2. Admixture analysis. The distribution diagram of the CV value, corresponding to the different K values.

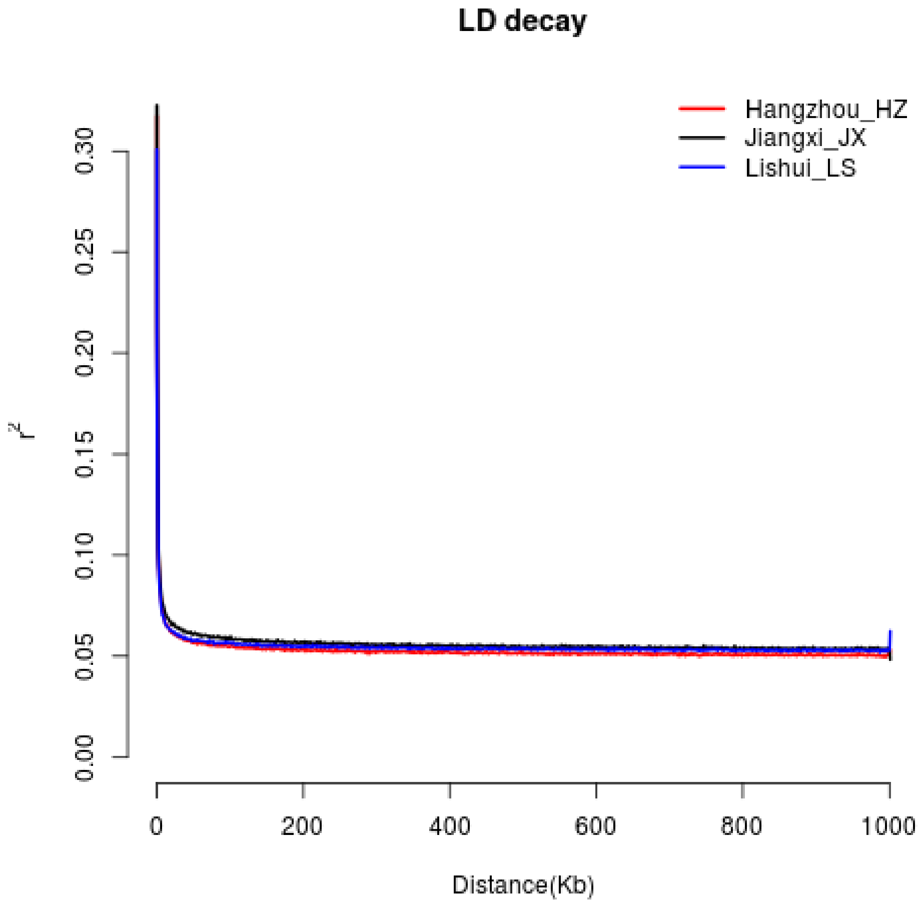

Figure S3. The distribution diagram of LD decay. The horizontal is the distance between SNP loci, and the vertical axis is the value of r^2. big value means strong LD decay, suggesting there frequently occurs inbreeding hybridization.

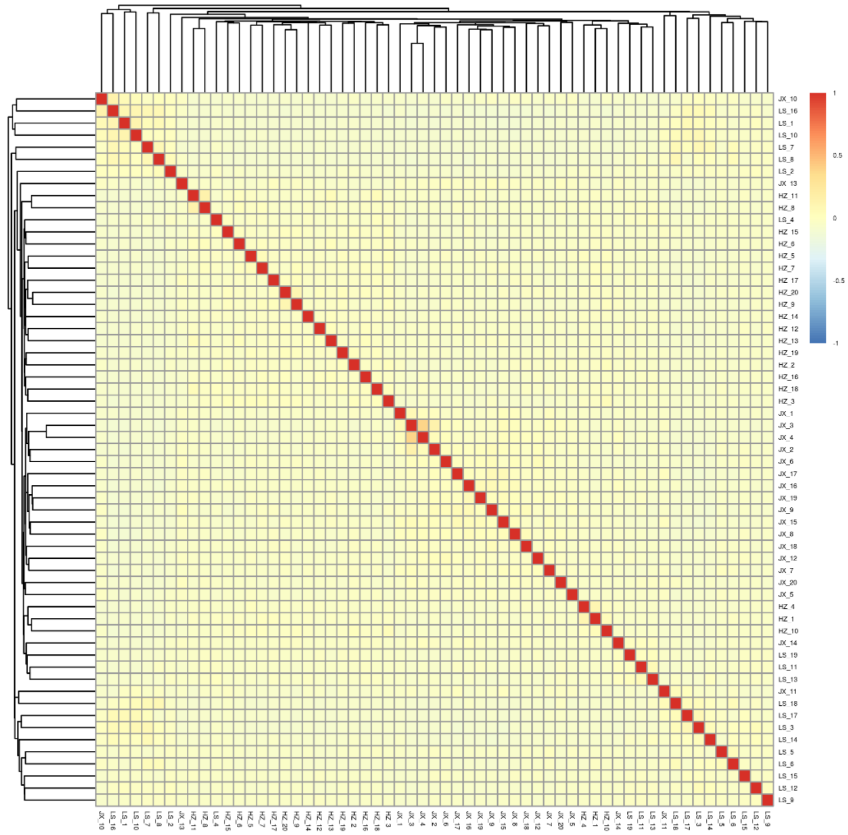

(a)

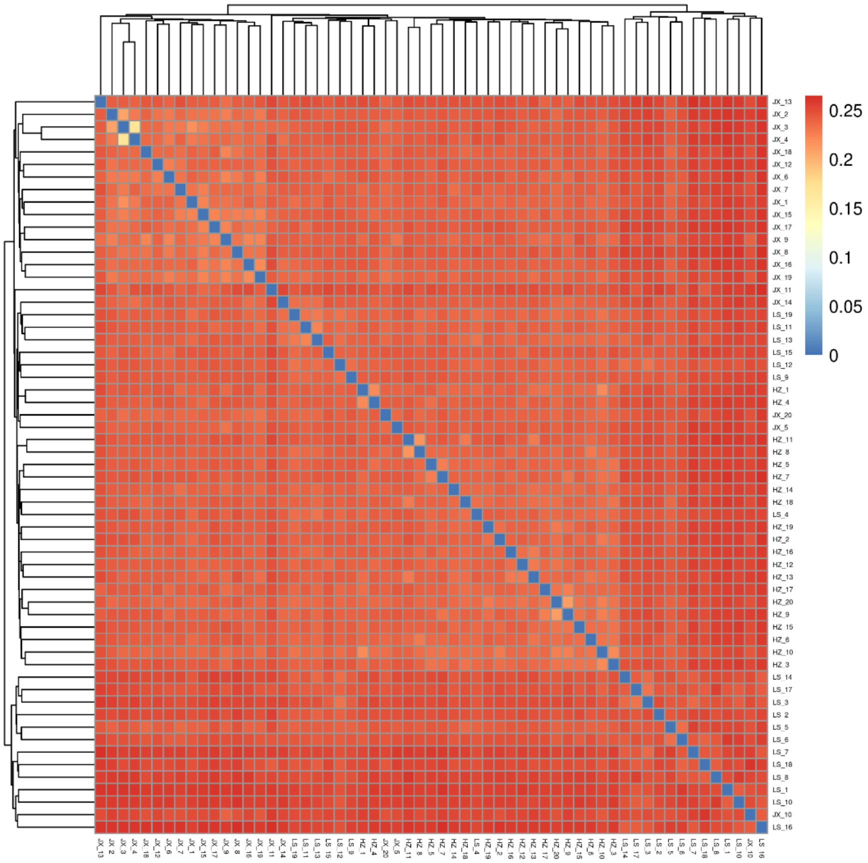

(b)

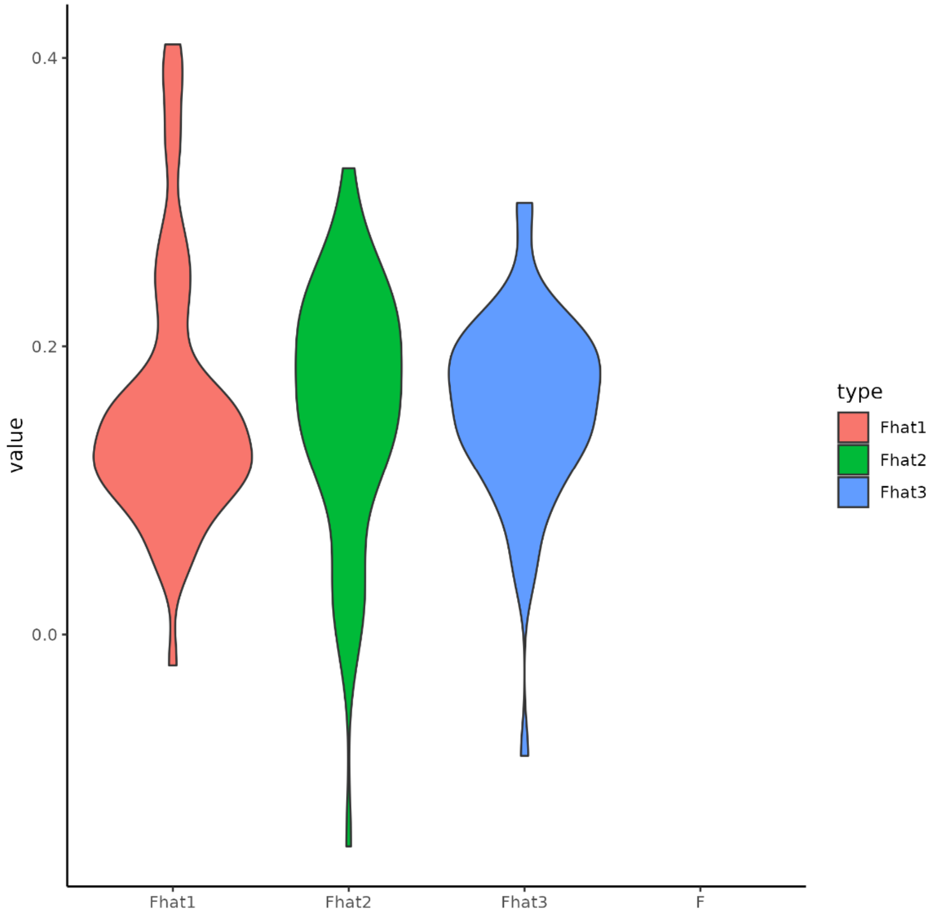

(c)

Figure S4. The genetic evolution of three subpopulations. (**a**) The heatmap of G matrix. The horizontal is sample ID, each block means the value of X-Y two samples. The color of blocks is close to red, with the big G value, meaning a closer relationship between the two samples from different subpopulation, and vice versa. (**b**) In the IBS matrix, blue blocks mean the short distance (lower differentiation and close relationship; red blocks mean long distance (higher differentiation and drift apart relationship. Corresponding to the G matrix, the three subpopulation has a closer relationship, converging altogether. However, the whole population representative to red, meaning higher differentiation exists among the samples, which facilitates the heterozygous hybridization. (**c**) The value of inbreeding coefficient.

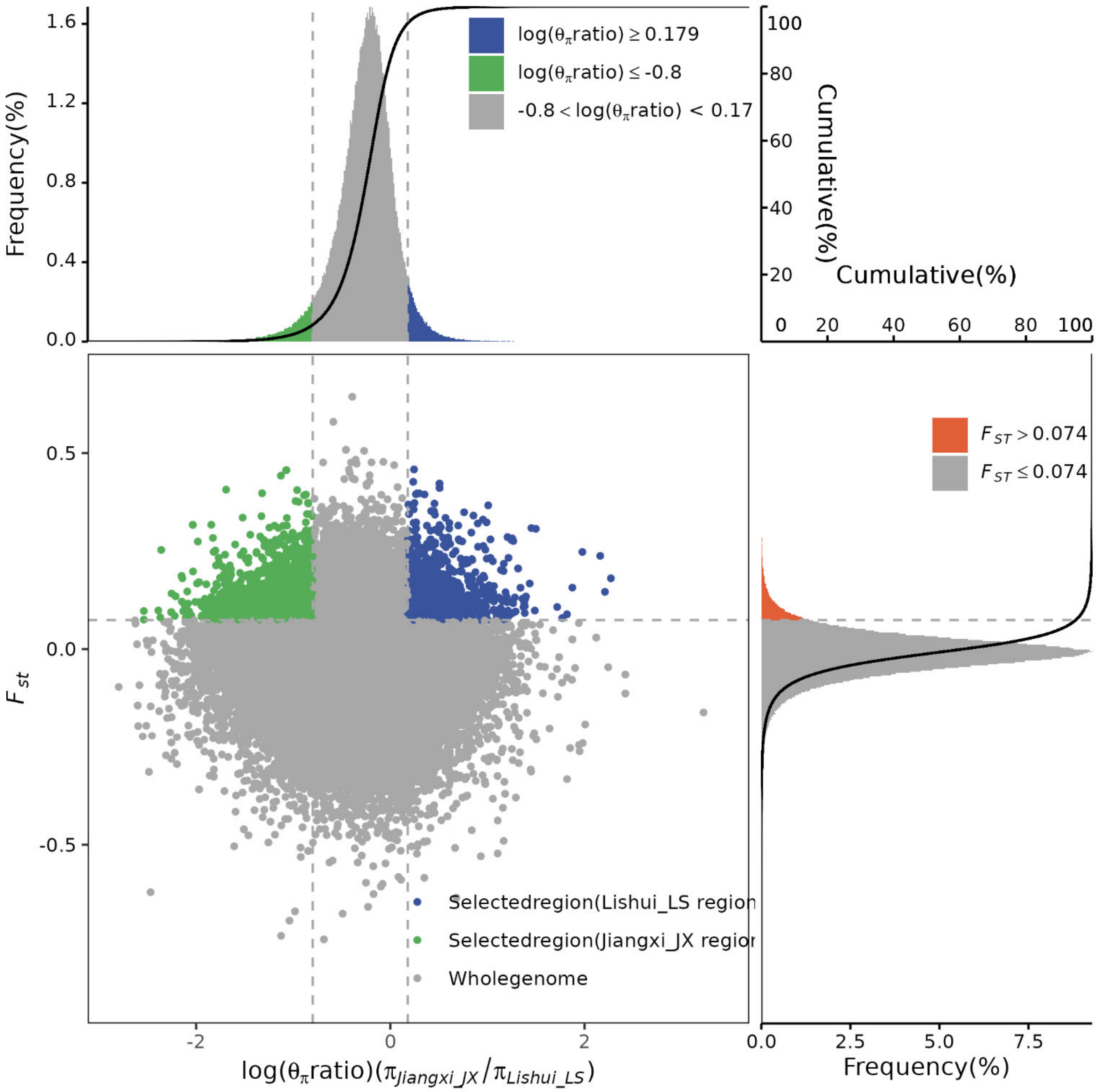

Figure S5. The plotted diagram of selective sweeping. The horizontal axis is the ration of θπ(sample)/θπ(control), and the vertical axis is the value of F_{st}; the different color is representative the unselective sweeping area (gray) and selective sweeping area (blue and red), respectively.

Table S1. SNP calling and mapping.

HZ_8	11,244,582	11,174,988	99.38%	0	0.00%
HZ_9	16,355,722	16,256,099	99.39%	0	0.00%
HZ_10	17,015,466	16,933,906	99.52%	0	0.00%
HZ_11	12,627,804	12,564,544	99.50%	0	0.00%
HZ_12	14,581,178	14,502,130	99.46%	0	0.00%
HZ_13	15,263,924	15,154,167	99.28%	0	0.00%
HZ_14	13,632,688	13,561,464	99.48%	0	0.00%
HZ_15	13,789,156	13,704,690	99.39%	0	0.00%
HZ_16	11,811,142	11,755,431	99.53%	0	0.00%

HZ_17	16,513,674	16,436,751	99.53%	0	0.00%
HZ_18	13,994,340	13,933,846	99.57%	0	0.00%
HZ_19	11,708,628	11,652,167	99.52%	0	0.00%
HZ_20	12,414,376	12,365,488	99.61%	0	0.00%
JX_1	16,158,580	16,084,068	99.54%	0	0.00%
JX_2	13,937,064	13,870,019	99.52%	0	0.00%
JX_3	11,644,974	11,589,717	99.53%	0	0.00%
JX_4	11,501,456	11,457,300	99.62%	0	0.00%
JX_5	13,177,860	13,124,440	99.59%	0	0.00%
JX_6	14,441,514	14,380,724	99.58%	0	0.00%
JX_7	13,057,270	13,009,954	99.64%	0	0.00%
JX_8	12,209,642	12,125,622	99.31%	0	0.00%
JX_9	15,518,198	15,425,932	99.41%	0	0.00%
JX_10	14,602,414	14,544,316	99.60%	0	0.00%
JX_11	15,039,988	14,979,759	99.60%	0	0.00%
JX_12	12,465,348	12,406,245	99.53%	0	0.00%
JX_13	7,880,144	7,842,685	99.52%	0	0.00%
JX_14	13,637,702	13,579,900	99.58%	0	0.00%
JX_15	15,221,052	15,148,512	99.52%	0	0.00%
JX_16	13,135,444	13,091,717	99.67%	0	0.00%
JX_17	15,168,598	14,731,901	97.12%	0	0.00%
JX_18	11,577,380	11,534,963	99.63%	0	0.00%
JX_19	17,867,026	17,780,025	99.51%	0	0.00%
JX_20	15,656,072	15,583,195	99.53%	0	0.00%
LS_1	12,398,674	12,346,349	99.58%	0	0.00%
LS_2	11,935,036	11,873,783	99.49%	0	0.00%
LS_3	14,110,850	14,053,722	99.60%	0	0.00%
LS_4	17,975,818	17,896,019	99.56%	0	0.00%
LS_5	14,936,018	14,872,152	99.57%	0	0.00%
LS_6	12,170,050	12,114,417	99.54%	0	0.00%
LS_7	18,775,266	18,676,018	99.47%	0	0.00%
LS_8	15,704,088	15,625,921	99.50%	0	0.00%
LS_9	16,040,282	15,959,209	99.49%	0	0.00%
LS_10	15,895,234	15,776,697	99.25%	0	0.00%
LS_11	12,242,268	12,132,348	99.10%	0	0.00%
LS_12	17,926,662	17,856,597	99.61%	0	0.00%
LS_13	17,570,464	17,488,728	99.53%	0	0.00%
LS_14	12,784,102	12,735,660	99.62%	0	0.00%
LS_15	12,094,142	12,038,260	99.54%	0	0.00%
LS_16	15,312,402	15,178,270	99.12%	0	0.00%
LS_17	20,244,176	20,160,490	99.59%	0	0.00%
LS_18	12,642,152	12,536,427	99.16%	0	0.00%
LS_19	16,781,506	16,648,565	99.21%	0	0.00%

59 samples mapping data

Sample	Total reads	Mapped reads	Mapping rate	Dup.nums	Dup.rate
HZ_1	12,780,156	12,708,924	99.44%	0	0.00%
HZ_2	11,910,256	11,833,806	99.36%	0	0.00%
HZ_3	14,172,176	14,103,260	99.51%	0	0.00%
HZ_4	11,536,880	11,482,810	99.53%	0	0.00%
HZ_5	13,533,746	13,470,653	99.53%	0	0.00%
HZ_6	16,399,960	16,317,934	99.50%	0	0.00%
HZ_7	12,200,600	12,143,321	99.53%	0	0.00%
